# Lek habitat suitability for the sharp-tailed grouse (*Tympanuchus phasianellus jamesi*) on the Northern Great Plains

**DOI:** 10.1371/journal.pone.0265316

**Published:** 2022-04-04

**Authors:** Brandon Burda, Christopher M. Somers, Katherine Conkin, Ryan J. Fisher

**Affiliations:** 1 Department of Biology, University of Regina, Saskatchewan, Canada; 2 Saskatchewan Ministry of Environment, Fish, Wildlife and Lands Branch, Saskatchewan, Canada; Zoological Survey of India, INDIA

## Abstract

Grassland birds in North America face many problems as a result of habitat loss and fragmentation; understanding their habitat requirements is critical for their conservation and management. The sharp-tailed grouse (*Tympanuchus phasianellus*) can be found throughout North American grasslands and is a species of economic and cultural importance, but it has experienced population declines over the last few decades. A large part of sharp-tailed grouse life history is focused on and around lekking grounds, which makes leks an essential feature for sharp-tailed grouse management. We used information from 596 leks and landcover predictors within 1-km and 5-km squares to perform Habitat Suitability Index modeling for sharp-tailed grouse on the Northern Great Plains in Saskatchewan, Canada. The proportion of grasslands at the 5-km scale and the 1-km scale were the two most important factors affecting lek occurrence (permutation importance = 34.8% and 26.9%, respectively). In every case, the 5-km scale predictors were ranked as having a more significant influence on lek occurrence than the 1-km scale. Other factors of importance included topographic roughness (9.7% permutation importance), and the proportion of human disturbance at the 5-km scale (5% permutation importance). Our study highlights the importance of large patches of grassland to support the occurrence of sharp-tailed grouse leks, and that a diverse set of habitat features are needed for sharp-tailed grouse management.

## Introduction

Temperate grasslands are considered one of the most endangered biomes on earth [1], with land conversion for agricultural use as the primary driver of grassland loss [2]. In Canada, only 25–30% of original grasslands still exist [3]. The quality of remaining grasslands is also affected by a variety of land uses that can decrease their habitat quality without necessarily destroying them. These land uses include unsustainable grazing, and energy extraction and distribution infrastructure needed to distribute energy from extraction sites, which can add additional anthropogenic features to the landscape including fencing, wells, turbines, transmission lines, and roads [3]. The presence, operation, and maintenance of these features and practices can create additional challenges for grassland species, including sensory disturbances and increased mortality [4–6]. Unfortunately, how these additional anthropogenic features and practices affect many grassland species is not fully understood.

Habitat specialists seem to be particularly at risk due to anthropogenic habitat changes, and the consequences of habitat loss and degradation are easier to predict for specialists compared to generalists [7]. The sharp-tailed grouse (*Tympanuchus phasianellus*) can be found in grasslands and in open areas within the boreal forest across North America [8]; however, due to its lack of preference for specific vegetation species communities within these open habitats, it has typically been classified as a habitat generalist [8]. Despite being classified as a habitat generalist, sharp-tailed grouse have experienced population declines in certain regions over the last few decades, particularly at the southern and western edges of their range [9, 10]. Habitat loss and fragmentation are likely principal factors causing the declines of these sharp-tailed grouse populations [9, 10]. However, part of the challenge associated with managing habitat of generalist species is that habitat use plasticity by these species may require region-specific research to inform conservation and management [8].

A typical unit used when managing sharp-tailed grouse and their habitat is known as the “breeding complex”, which is focused on their mating grounds (hereafter, leks) and typically a 2-km radius around the leks [8, 11]. Leks are communal display arenas where males compete for females; males show high site fidelity to the same lek area over the years [12]. Sharp-tailed grouse home ranges change throughout the year, but males spend the majority of the year in close proximity to the lek site [8, 9, 13]. Once a female has mated she will typically travel 1–2 kms away from the lek to nest [14, 15]. Due to the central role of leks in the breeding complex and the high detectability of male displays, leks are excellent units for the study of sharp-tailed grouse habitat use and subsequently, management [11]. Understanding what features sharp-tailed grouse select at and around lek sites can help delineate important habitat. However, due to the variability of habitats and landscapes used by the seven sharp-tailed grouse subspecies [8], the consistency of features selected for lekking may differ within each subspecies range.

Here we examined habitat features influencing lek occurrence for the plains sharp-tailed grouse (*T*.*p*. *jamesi*) in southern Saskatchewan, Canada. The plains sharp-tailed grouse inhabits primarily shrubby grasslands across the Great Plains of North America, and their leks are usually found in areas with short grasses, often associated with shrubs, on natural rises [10, 16, 17]. Female grouse require the presence of tall vegetation or woody shrubs for nesting and brood-rearing, though these features are typically found within larger open grassland complexes [18]. Nesting habitat contrasts with lek sites, whereby leks may be abandoned if too much woody vegetation is accumulated [19]. Fine-scale models that include features such as vegetation height exist for predicting suitable plains sharp-tailed grouse habitat, but these models focus primarily on nesting and brood-rearing habitat [20]. Areas with tall residual grasses are considered optimal for sharp-tailed grouse nesting and brood rearing, whereas approximately 5–10% shrub cover is considered optimal for wintering habitat [20]. However, the outcomes of these fine-scale modeling efforts are often difficult or impossible to extrapolate over a large area to predict sharp-tailed grouse habitat and manage it appropriately.

Our study used a combination of field surveys and remotely sensed landcover and topographic data to examine multiple habitat factors that are predicted to influence sharp-tailed grouse lek occurrence. We acquired lek information using contemporary (2018–2019) and historical field surveys (1990–2004), government pre-development surveys (1995–2016), and citizen science data (2017–2019) in the prairie ecoregion of Saskatchewan. As a generalist species, sharp-tailed grouse are less likely to be reliant on particular vegetation species, so we focused on using coarser land cover-based habitat assessments. These landscape variables are easy to monitor and acquire at large scales, making this type of analysis easier to apply for decision making over large landscapes. We modelled of the suitability of habitat for leks across the grasslands of southern Saskatchewan, Canada using a Habitat Suitability Index (HSI). Based on the HSI, we determined the relative rankings and importance of different habitat features for predicting the suitability of a site for leks. Lek sites are only one part of the breeding complex, so we predicted that leks would be influenced more by their surrounding habitat features than those directly at the lek location.

## Methods

### Study area

Our study area encompassed the prairie ecozone in southern Saskatchewan, Canada (latitude range 49 to 52° and longitude range -101 to -110°) and included an area of approximately 240,967 km^2^. Southern Saskatchewan receives an average of 395 mm of precipitation per year, with most falling in the month of June, and the average maximum temperature in the summer is approximately 25.5°C [21]. The study area consists of two major land-uses, annual crop production and grazing by domestic cattle, with an extensive road network and other smaller land-uses including oil and gas extraction, urban and rural habitations, and mining. Our study area is in the northeastern range of the plains subspecies of sharp-tailed grouse [8].

### Historical surveys

The Saskatchewan Ministry of Environment (ENV) conducted sharp-tailed grouse lek surveys across the prairie and parkland ecoregions of Saskatchewan from 1958 to 2004. These surveys were used to develop the framework for our survey protocols and locations. These historic surveys were conducted within typically township-sized (approximately 41.4 km^2^) predefined survey blocks that included a mix of grazed grassland and annual cropland (two of the dominant land uses in Saskatchewan). Driving surveys were conducted in April and May from one hour before sunrise until two hours after sunrise on days without rain and wind speeds below 25 km/h. Stops were conducted every 1.6 km (1 mile) for a minimum of two minutes to listen and visually survey for leks. Once a lek was discovered, surveyors recorded the location as either a quarter-section (a 800m x 800m unit of land division) or a GPS location once this technology became widely available. Counts of both male and female grouse in attendance were conducted, and if possible a flush count, in which birds were made to fly away from the lek, was also done. Each block was surveyed twice within a season when possible. Not all blocks were surveyed consistently throughout the program, and new blocks were added as the program matured.

### Contemporary surveys

Contemporary field surveys were conducted similarly to the historical ones performed by ENV. We used the same field protocols with a few modifications. The surveys were shortened to start 30 minutes before sunrise to aid visibility and to ensure that birds were attending leks. In our first few surveys, birds at many leks did not become active until around 45 minutes before sunrise, with males continuing to arrive over the next few minutes. Flush counts were not conducted as we did not want to disturb the grouse, and in turn it reduced our time spent at each lek and allowed us to cover a larger geographic area. We began our surveys on March 26^th^ in 2018 and March 25^th^ in 2019 and continued until June 1^st^ for both years. We had two teams, each of which surveyed each block once, for a total of two surveys per block. We surveyed both historical (18 in 2018 and 14 in 2019) and new survey blocks (10 in 2018 and 22 in 2019) for a total of 57 blocks surveyed. Surveys initially covered as many accessible roads as possible within each block; the second set of surveys was used to resurvey identified leks to confirm occupancy and counts.

We conducted driving surveys stopping every 1.6 km to look and listen for lekking grouse. The low cooing calls males make on the lek can be heard from up to four km [22], with the farthest lek heard during our field seasons being approximately 2.6 km away from a stop point. We also continued to look for birds between stops. At every stop, we recorded the location (GPS point), any signs of sharp-tailed grouse (leks, calls, or birds observed), and the surrounding habitat (native grassland, tame grassland, or cropland). When a lek was located we observed it for a minimum of ten minutes to ensure all birds were counted. In addition, we also recorded the behaviour of the grouse (dancing, calling, or inactive) and the habitat surrounding the lek.

### Other lek location information

Additional lek locations were supplied by the Saskatchewan Conservation Data Centre (CDC; 1995–2016) and the Saskatchewan Ministry of Environment’s Co-operative Wildlife Management Survey App (CWMS; 2017–2019). These additional tools allowed citizen scientists and environmental contractors to report lek locations to us across our study area. The CDC quality assures and controls observational data using NatureServe protocols [23]. To ensure the quality of the CWMS leks, all records we used were limited to those made in April and May mornings and where dancing behaviour was recorded. By adding additional lek locations from these programs we acquired a better geographic representation of lek locations (i.e., lek habitats and surrounding landscape composition) across our large study area for training the model. In addition to the datasets that we used for model training purposes, we also gathered independent data from eBird on sharp-tailed grouse leks in Saskatchewan. eBird data was filtered to match datasets used above: observations had to occur in the last five years (2017–2021), ≥ 3 grouse were observed, and observations were limited to April and May.

### Habitat suitability index

We used MaxEnt to create the Habitat Suitability Index for sharp-tailed grouse in Saskatchewan. This approach is typically competitive or outperforms other modeling methods for predictive accuracy when using presence-only data [24, 25]. Our data partially consisted of standardized presence and absence surveys (i.e., historical and contemporary surveys); however, incorporation of the citizen-science presence-only data necessitated the use of this type of analysis. MaxEnt requires two primary pieces of data to create the habitat model: known occurrence data for leks, and rasterized maps of environmental predictor variables (habitat features; see below). Due to potential changes in land use over time, we limited use of historical lek data from the ENV surveys to the period since 1990. This decision was made to produce the best possible balance between retaining lek data, and matching the years represented by the environmental predictor layers (see below). Field data from 2018–2019, as well as lek occurrence information from the CDC (1995–2016) and CWMS app (2017–2019) were also used. We only included leks in our analyses if there were three or more males displaying (dancing, calling, territorial displays, or mating; [26]). As lek locations were used for the presence data in the model, the model is more correctly an index of lek occurrence and not necessarily reflective of sharp-tailed grouse habitat throughout the yearly life cycle (e.g., brood-rearing, wintering habitats). Although sharp-tailed grouse occupy all of Saskatchewan, we limited the background (geographical range) for MaxEnt modeling to the prairie ecoregion of Saskatchewan (240,967 km^2^) given that this was where all of our lek location data came from. The Maxent algorithm compares locations of where a species is present to 10,000 random samples of available habitat within the geographical range we defined above.

### Environmental predictor variables

**Fig 1:**
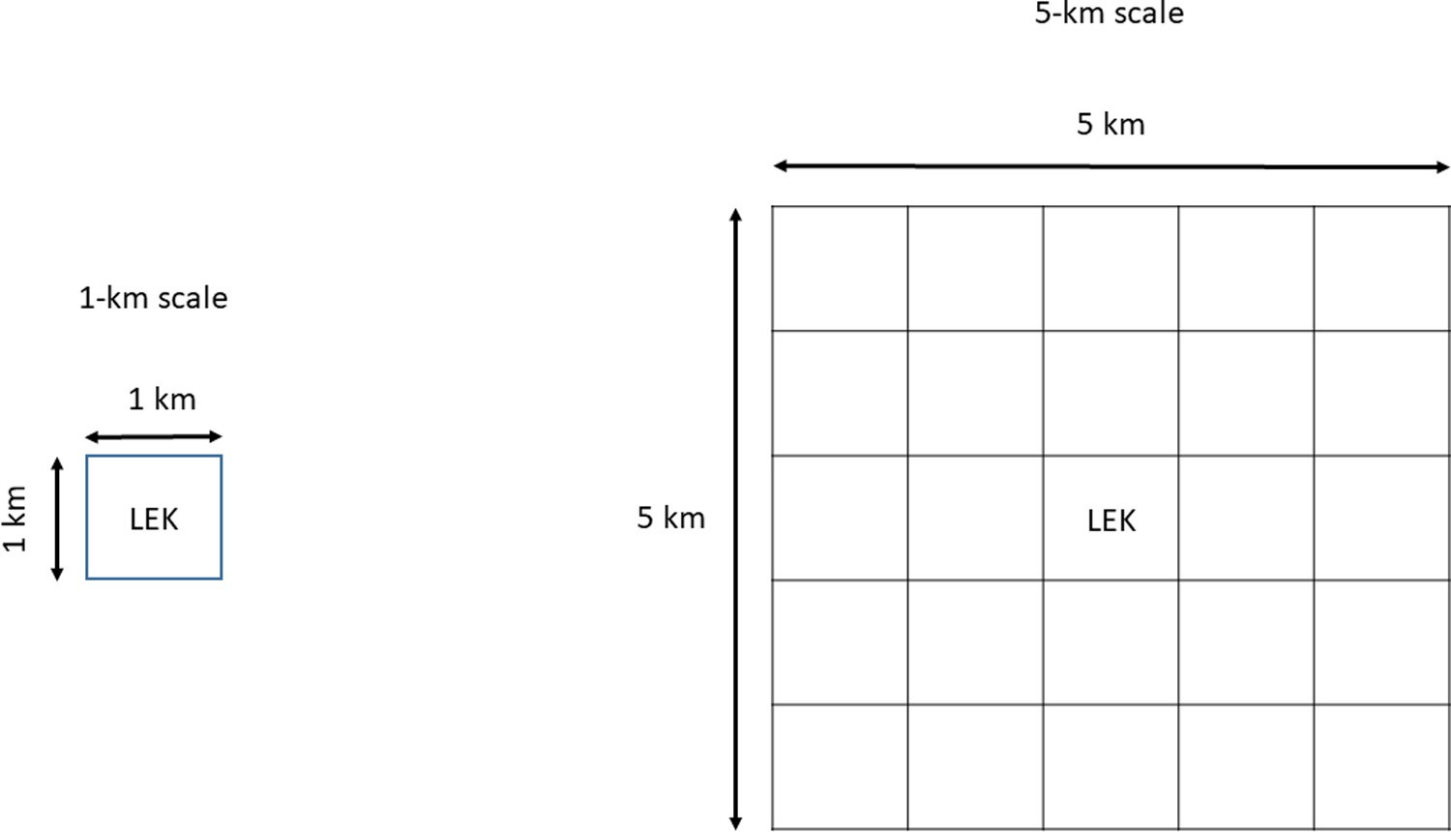
Schematic representation of the scales at which environmental predictor layers were measured around each lek.

Two types of environmental predictor layers were created for use in our model: proportions of various landcovers and a terrain roughness index. All the layers had a pixel size of 1 km x 1 km. This resolution was chosen so that our results could be incorporated and matched to current large-scale mapping and management efforts in the province of Saskatchewan and because some lek locations had only a quarter-section identification rather than an exact GPS location. The proportion of landcover layers were created using 2010 land use maps created by Agriculture and Agri-Food Canada [27], which was the most recently available and reliable landcover layer for the province of Saskatchewan. These land use layers have a 30-m resolution, so it should be noted that smaller features, such as power poles and smaller patches of habitat, are not mapped by this product. In ArcMap 10.6 (ESRI), we converted this raster layer to vector format, a 1-km x 1-km grid was then overlaid on the resulting shapefile, and the proportions (0–1) of each landcover type (Fig 1) within each 1- x 1-km pixel were calculated using the Tabulate Intersection tool (Fig 1). We also calculated the proportion of each landcover type within a 5 x 5-km square with the lek at its center (i.e., roughly equivalent to the 2-km radius breeding complex [11]; Fig 1). Grasslands are a landcover dominated by graminoids and forbs. The amount of grassland in the landscape is inversely correlated with cropland and so we selected grassland to be included in our modelling rather than both dominant landcovers. Shrub landcover is typically woody vegetation ≤2-m tall and the tree landcover represents areas with vegetation ≥2-m tall. The tree landcover is typically limited to river valleys and some small forested areas, representing <5% of the study area. Wetlands are typically small permanent or ephemeral bodies of water and water represents large bodies of water such as lakes and rivers; however, wetlands can sometimes include larger more permanent waterbodies. Exposed earth represents areas with naturally occurring exposed rock or soil. The human disturbance landcover represents areas where vegetation has been impacted by human activities (e.g., roads, parking lots) and areas with human structures (e.g., buildings) and was based on the “built-up” category in the AAFC dataset. The terrain roughness index layer (hereafter, roughness) was created using AdaptWest’s Land Facet Data for North America [28] using the R package “raster” [29] on the eight neighboring pixels around each lek pixel (i.e., a 3 x 3 pixel square around the lek) and was only examined at the 1km x 1km scale.

### Statistical analysis

As a result of our presence-only points being clumped due to focused survey efforts, we first rarified the lek data to reduce sampling bias and potential issues with spatial autocorrelation [30, 31]. Using SDMToolbox v. 24 [32], points were reduced to a single record if they were within 5 km of one another, this distance was selected because: (1) it prevented overlap between environmental covariates at the 5-km x 5-km scale, (2) it should reduce the impacts on AUC scores of training and testing data points that are close to one another, (3) this is well outside the typical 1–2 km distance that females travel to nest away from a lek, and (4) we found several leks that were within 2-km of each other, suggesting that any lek selection outside the 5-km distance is likely independent. A biased sampling effort can also have negative consequences on models produced from Maxent [33] so we constructed a bias grid that allowed us to upweight sample points with fewer neighbours [34]. This bias grid was constructed in SDMToolbox v 2.4 [34] using the Gaussian Kernel density of Sampling Localities algorithm and a sampling bias distance of 50 km. A value of 1 in the output bias grid indicates no sampling bias and values larger than 1 indicating some degree of sampling bias [34].

Given that our study occurred over a large area and included multiple ecoregions and ecozones, we used the spatial jackknifing (i.e., geographically structured k-fold cross-validation) approach in SDM Toolbox v. 2.4 with three spatial groups for modelling [32, 35]. Models are calibrated with k-1 spatial groups and then evaluated on a holdout region. In a case with three spatial groups (X,Y, and Z), the model is calibrated using presence and background points from region X and Y and then evaluated on points from region Z, then calibrated using presence and background points from region Y and Z and then evaluated on points from X and so on.

We also conducted independent tests on the model feature class (Table 1) parameters which allowed us to test different combinations of five model feature types available in Maxent and select the best combination: (1) linear, (2) linear and quadratic, (3) hinge, (4) linear, quadratic and hinge, and (5) linear, quadratic, hinge, and product. We used 25 replicate models and cross-validation to generate average AUC scores for each of these model feature type combinations. The models were then ranked such that the model with the lowest omission rate was considered the best, but if any models were tied, then the model with the highest AUC was selected as the best model [35]. We constructed 25 replicate models using cross-validation to generate the AUC score for each spatial area and the overall final model. Predictive ability for the final model was assessed using the area under the curve (AUC) for both the training and testing datasets. In addition to using AUC, we also used the ecospat package in R [36] with a spearman correlation to asses the Continuous Boyce Index (CBI; [37]) on the independent dataset from eBird. The CBI is a thresholdless metric that only requires presences and measures how much model predictions differ from the random distribution of the observed presences across the prediction gradients [37]. The CBI varies between -1 and 1, where positive values indicate the predictions for presences are consistent with the distribution of presences in the evaluation dataset, values close to 0 indicate that the model is not different from a random model, and negative values indicate opposite predictions (predicting poor quality areas where presences are more frequent). Individual predictor layer importance was assessed by the permutation importance averages. The permutation importance is based on the drop in training AUC from a re-evaluated model using randomly permutated data between the environmental predictor and training presences and background data [24]. We did not convert the resulting predictive raster into binary suitable and unsuitable habitat polygons because there are likely multiple uses for our final predictive rasters that may benefit from selection of different threshold values; furthermore, it is recommended that using a continuous scale of prediction avoids undue confidence in binary suitable/unsuitable habitat designations [24].

**Table 1:** Model selection for feature class parameters using the spatial jackknife approach in Maxent. Models with the lowest omission rate was considered the best model.

Feature parameters	Regularization Multiplier	Omission Rate	AUC
Linear, Quadratic and Hinge	1	0.604	0.624
Linear, Quadratic	1	0.627	0.580
Hinge	1	0.629	0.633
Linear	1	0.648	0.544
Linear, Quadratic, Hinge, and Product	1	0.707	0.592

## Results

### Lek surveys

**Fig 2:**
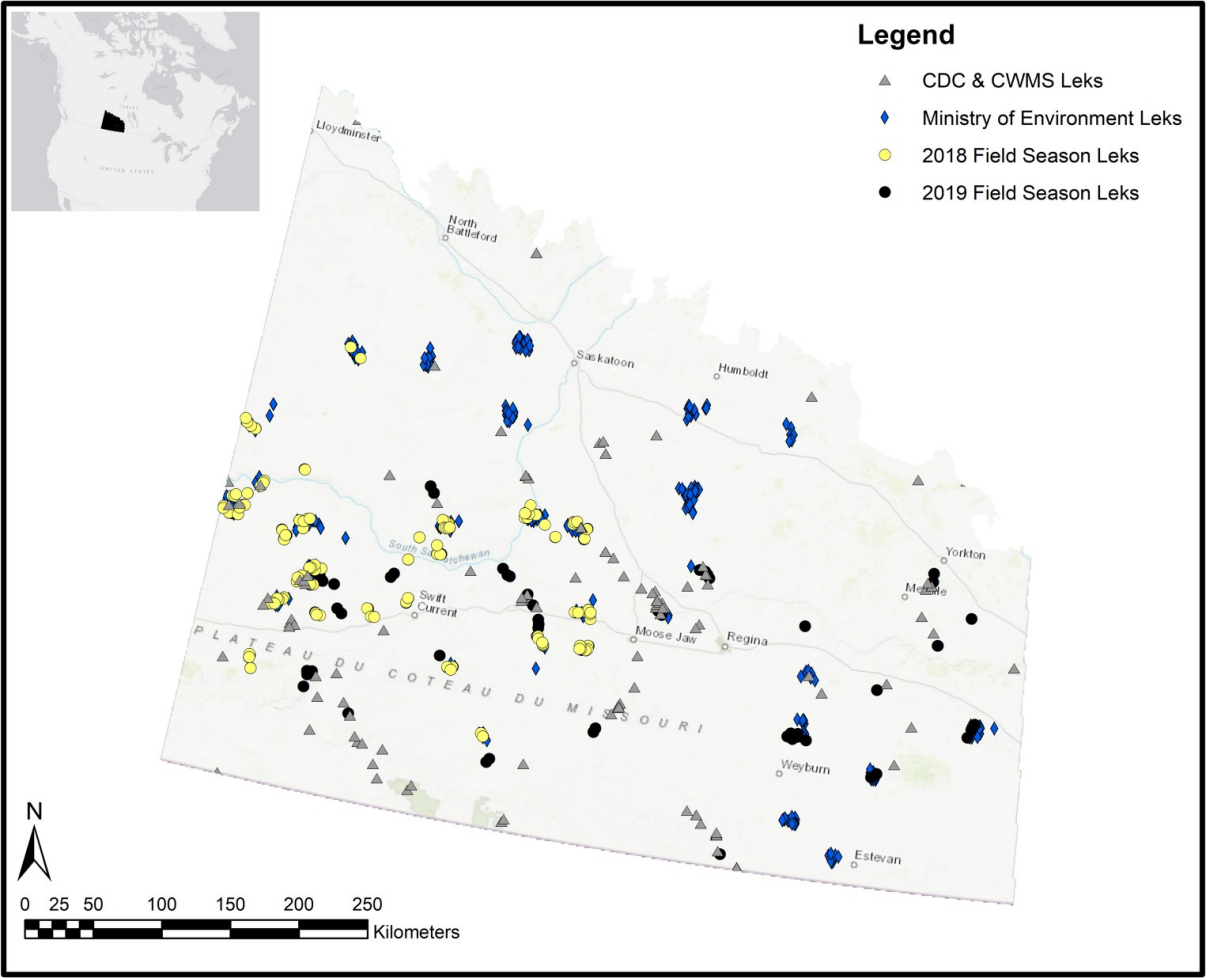
Location of sharp-tailed grouse leks discovered in our contemporary field surveys and other sources in southern Saskatchewan, Canada. Leks located in 2018: yellowcircles; Leks located in 2019: black circles; CDC and CWMS leks (1996–2016): grey triangles; and, Ministry of Environment leks (1990–2004), blue diamonds.

**Fig 3:**
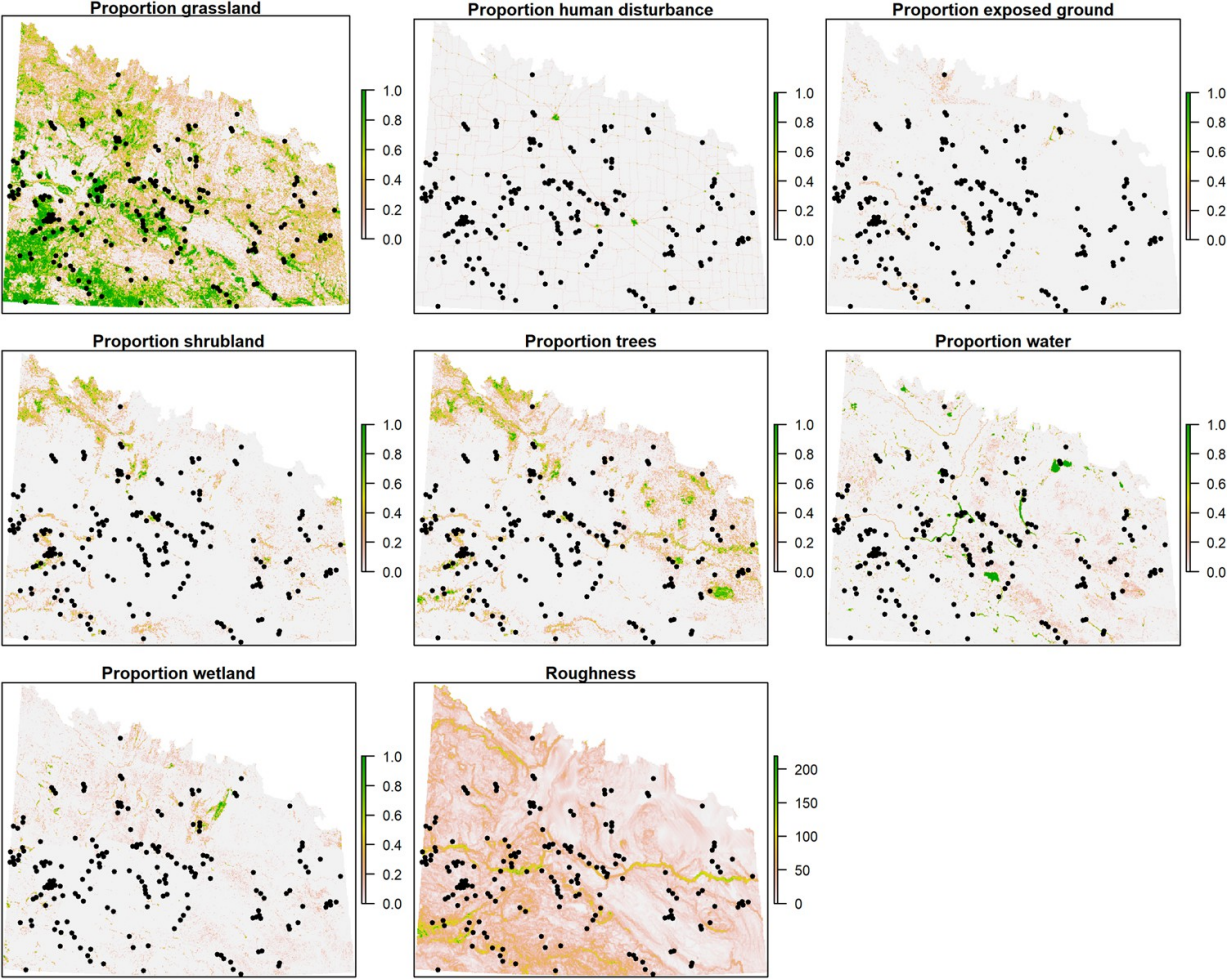
Locations of rarified sharp-tailed grouse leks in Saskatchewan, Canada that were used in our analyses and 8 environmental predictors at the 1km x 1km scale (5- x 5-km scale not shown). Leks are shown as black circles.

We located 147 leks during contemporary field surveys, 87 in 2018 and an additional 60 in 2019 (Fig 2). Of those 147 leks, only 28 were previously recorded during historical ENV surveys. The leks were detected on 57 survey routes, of which 25 routes were the same as historical ENV surveys. On six of the 57 (10.5%) survey routes no leks were recorded. In total, we had access to 596 lek locations, 104 from the CDC, 30 from the CWMS, 147 from current surveys, and 315 from the historical ENV surveys (Fig 2). After spatial rarefaction, we used 185 leks for our HSI modelling purposes to test for the effects of the different environmental predictors on lek occurrence (Fig 3). For each of the three spatial jackknife processes, the sample sizes for training data in each were 59, 117, and 103 respectively and for testing, samples sizes were 19, 38, and 34, respectively. For training the final model across the entire study area, a sample of 139 leks was used and 46 leks for testing.

### Habitat suitability model and predictor rankings

**Fig 4:**
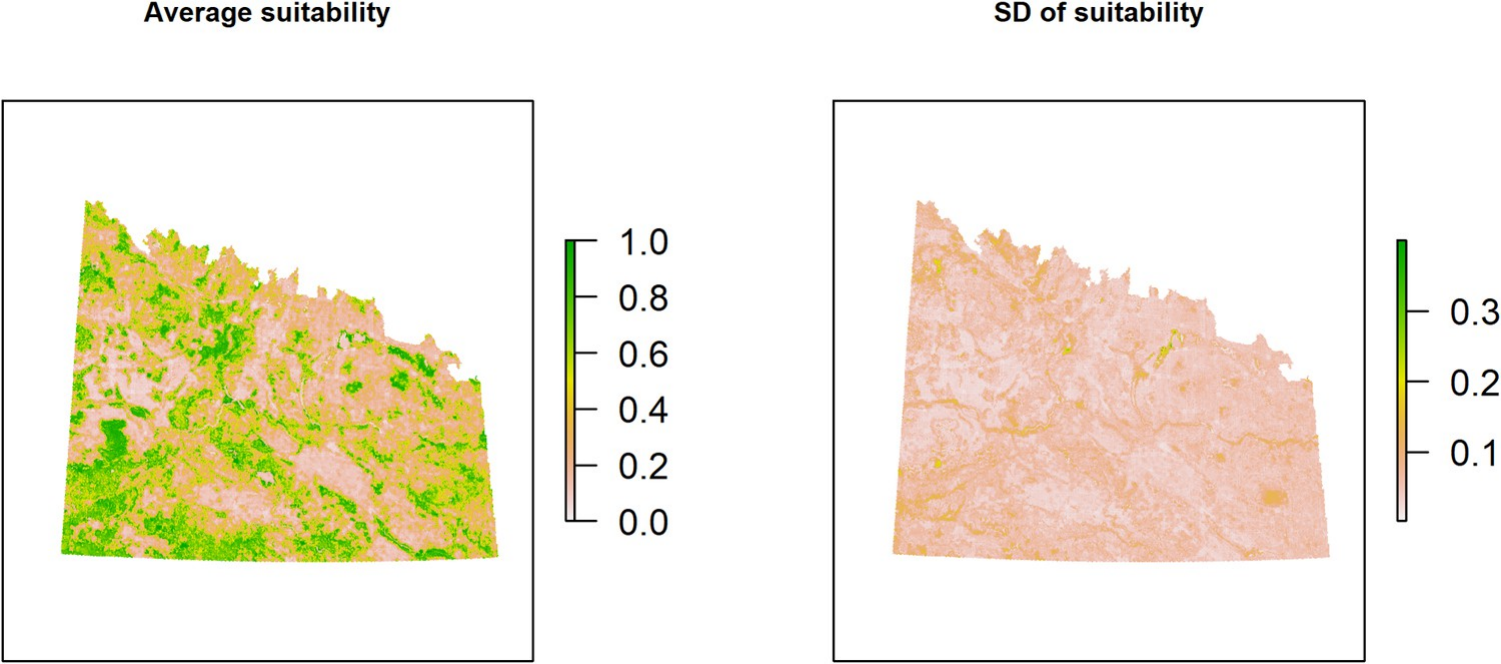
MaxEnt model-predicted probability of occurrence of sharp-tailed grouse leks across southern Saskatchewan, Canada. The model is based off the average of 25 cross-validation runs of the final model and the standard deviation (SD) of those 25 runs is also presented.

From the spatial jackknife process, a model with linear, quadratic and hinge features with a regularization multiplier of 1 was selected as the best model with the lowest omission rate, highest AUC, and least complexity (Table 1). The final model (Fig 4) had a training AUC of 0.803 and a testing AUC of 0.748 (± 0.032 SD) and predictions of lek habitat suitability were made across the study area (Fig 4). The CBI for the independent eBird dataset (N = 77 leks) was 0.714 indicating the predictions for presences are consistent with the distribution of presences in the eBird dataset. For every predictor variable, the 5 x 5 km scale had a higher permutation importance compared to the 1 x 1 km scale (Table 2). The proportion of grassland at the 5 x 5-km and 1 x 1-km scales were the most important variables for predicting lek occurrence, with a combined permutation importance of 61.7 (Table 2). Roughness was the next most important variable (permutation importance = 9.7), followed by the amount of human-caused disturbance at the 5- x 5-km scale (permutation importance = 5.2), the amount of trees at the 5- x 5-km scale (permutation importance = 4), and the proportion of wetlands at the 5- x 5-km scale (permutation importance = 3.3). All other predictors had a permutation importance <3% (Table 2).

**Table 2:** Permutation importance and percent contribution of environmental predictors for the final average MaxEnt model.

Variable	Permutation importance	Percent contribution
Proportion of grassland 5 x 5 km	34.8	31.1
Proportion of grassland 1 x 1 km	26.9	46.3
Roughness	9.7	4
Proportion of human disturbance 5 x 5 km	5.2	1.7
Proportion of trees 5 x 5 km	4	4.4
Proportion of wetlands 5 x 5 km	3.3	1.5
Proportion of wetlands 1 x 1 km	2.8	2.1
Proportion of exposed earth 5 x 5 km	2.7	2.5
Proportion of water 5 x 5 km	2.7	0.7
Proportion of human disturbance 1 x 1 km	2.5	0.5
Proportion of water 1 x 1 km	1.9	1.6
Proportion of shrubs 5 x 5 km	1	0.8
Proportion of exposed earth 1 x 1 km	0.9	1.2
Proportion of trees 1 x 1 km	0.9	0.9
Proportion of shrubs 1 x 1 km	0.7	0.5

### Relationship between major predictors and the probability of occurrence

**Fig 5:**
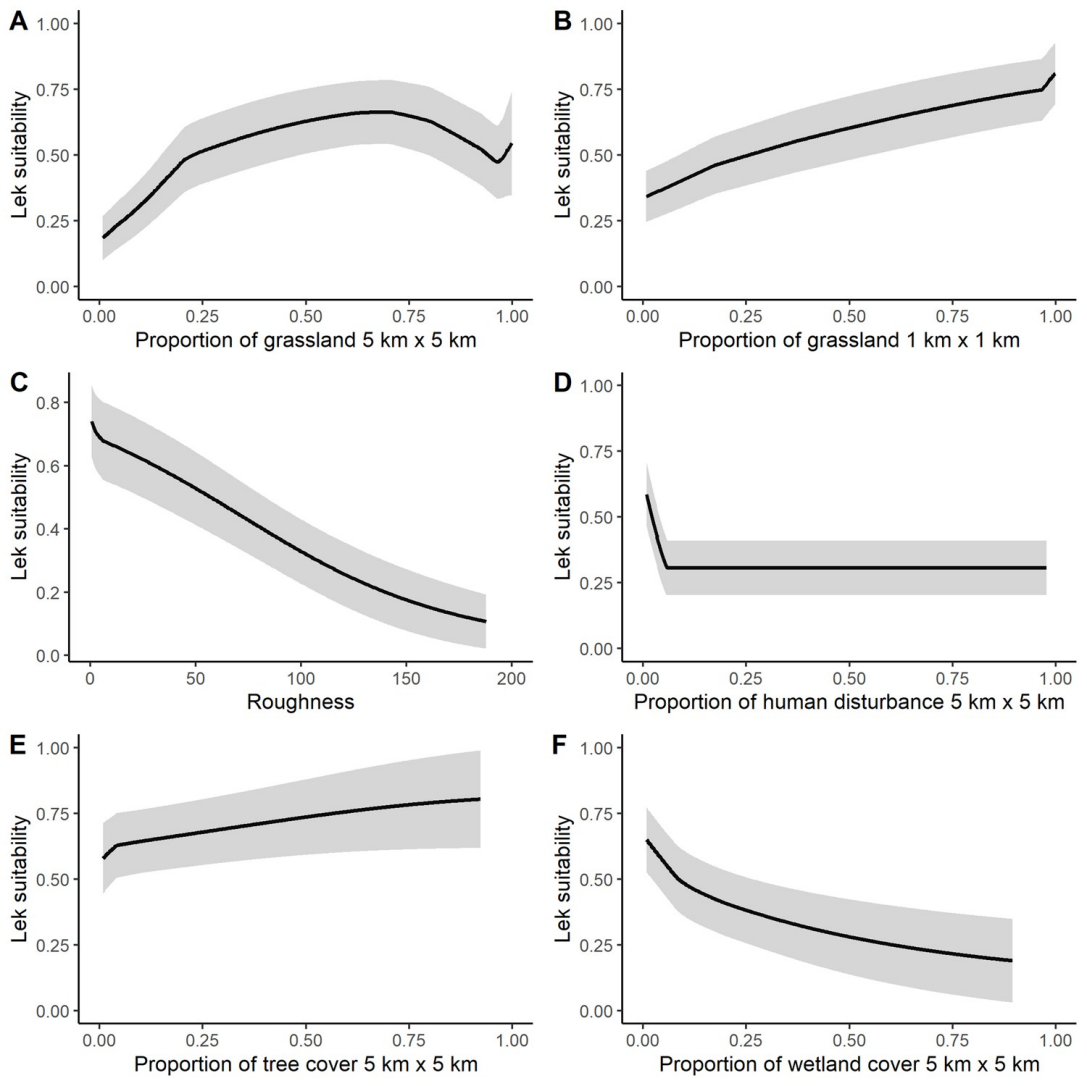
Model-predicted probability of lek occurrence responses curves for environmental covariates that had permutation important of >3%. A) Grassland cover at the 5 x 5-km scale, B) Grassland cover at the 1 x 1-km scale, C) roughness, D) Human disturbance at the 5 x 5-km scale, E) Tree cover at the 5 x 5-km scale, and F) wetland cover at the 5 x 5-km scale. The line is the average response curve over the 25-model cross-validations and error bars are± 1 SD.

Habitat suitability increased with the proportion of grasslands in the 5 x 5-km scale surrounding the lek, but started to decrease when the proportion of grassland reached approximately 0.75 (Fig 5A). In contrast, the 1 x 1-km scale grassland predictor (Fig 5B) showed a simple positive relationship with lek occurrence. The next most important predictor was roughness (Fig 5C), which represents the topographic condition around the leks. Areas with lower roughness were more favorable for lek occurrence. From our own field surveys, it was apparent that leks were often on small hills and rises in relatively flat landscapes. The next most crucial environmental predictor was the proportion of the anthropogenic built-up area at the 5 x 5-km scale (Fig 5D). The suitability of a site dropped sharply between 0–0.05 proportion built-up area and 5- x 5-km areas with greater than 0.05 proportion of anthropogenic disturbance had low suitability. The proportion of tree cover at the 5 x 5-km scale showed a positive relationship with lek suitability (Fig 5E). Lastly, the proportion of wetlands at the 5 x 5-km scale (Fig 5F) was the fourth-best predictor. Habitat suitability was highest in 5 x 5-km areas with wetland cover from 0–0.1, but otherwise 5 x 5-km areas with high wetland cover were not suitable (Fig 5F). None of the other variables contributed meaningfully to the model (Table 1).

## Discussion

Overall, our sharp-tailed grouse lek occurrence model for southern Saskatchewan, Canada was robust and provided a high degree of predictive accuracy (i.e., AUC scores indicated good predictive ability on the testing and training datasets and our model had a high CBI on a completely independent dataset). Our use of coarse remotely sensed land use information adequately captured broad patterns of sharp-tailed grouse lek occurrence, even though sharp-tailed grouse are typically identified as a habitat generalist [8]. Finer-scale remotely sensed products and detailed habitat measurements (e.g., vegetation height) could help to refine our models further; however, our results still provide helpful information for decision-makers on lek and surrounding habitat needs of sharp-tailed grouse. Furthermore, the scale at which our model was constructed is well-aligned with the land cover mapping products available in many jurisdictions across North America. As updated mapping products become available, wildlife managers should be able to reassess the amount of sharp-tailed grouse habitat.

For all environmental predictors, the larger 5 x 5-km scale predictors were ranked higher than the 1 x 1-km scale predictors. This finding is consistent with other studies that found habitat features within the breeding complex radius were more influential than habitat features where the lek was located [10, 38]. It has been suggested that a minimum area of 40–56 km^2^ of intact suitable habitat is required to support the minimum viable populations of sharp-tailed grouse [39, 40]. Individual grouse use much smaller areas but still require relatively large intact patches of habitat, averaging 6.17 km^2^ for males and 4.64 km^2^ for females [8, 9]. As both male and female grouse will spend most of their time around lek sites during the breeding season, leks are expected to be positioned in, and surrounded by, suitable year-round habitat. Outside of the daily needs of the grouse (feeding, cover), the most important use of habitat around the leks is for nesting and it has been suggested that leks rarely persist unless suitable nesting habitat is nearby [41]. The high importance of grasslands at both the 1 km- and 5 km-scales in our study is consistent with the results from these previous studies [41].

The importance of the 5 x 5-km grassland predictor highlights the need for large areas of intact grassland for sharp-tailed grouse, similar to other previous studies [42, 43]. In the United States of America, the Conservation Reserve Program (CRP) has provided an incentive for marginal cropland to be reseeded and maintained as grassland. In 10 of the 12 states where both sharp-tailed grouse and the CRP program exist, the sharp-tailed grouse range has expanded and populations have increased [44]. The greatest benefits were found when the CRP stands were located next to pre-existing grouse habitat, in effect augmenting and increasing the size and diversity of those grasslands [44]. For both sharp-tailed grouse and other prairie grouse simply having grasslands, especially in a monoculture, is not necessarily beneficial; rather, diverse grasslands with a variety of different vegetation types increased the habitat quality considerably [44]. Maintaining large, intact grasslands is especially important in the northern part of the sharp-tailed grouse range where this landcover type has been reduced to less than 30% of historical levels [3].

While grasslands were important for predicting sharp-tailed grouse lek occurrence, leks were typically surrounded by other habitat features. The habitat of plains sharp-tailed grouse is often described as either subclimax brush or shrub-grassland [45], with previous research highlighting the importance of having a low percentage of shrubs and other sources of cover present [20, 46, 47]. Our model also highlighted that tree-cover was important for predicting lek occurrence at the 5 x 5km-scale. While the importance of a high amount of tree cover may be counterintuitive since sharp-tailed grouse need open areas for lekking, these areas with high tree cover may be important in the non-breeding season (i.e., overwintering) for feeding, roosting, and cover from predators [8]. Furthermore, these areas with high tree cover are some of the only remaining natural habitats on this anthropogenic landscape and typically occur within protected area boundaries making them critical areas for the species. We also found that the probability of lek occurrence decreased with increasing wetland cover.

The exact placement of the lek is defined by more than just landcover; for example, lek occurrence may also depend on topography [8, 14]. Our modelling suggested that sharp-tailed grouse preferred to lek in flatter areas than the surrounding landscape at the 1km x 1km scale. Leks as display grounds rely on visibility and detectability to be seen by passing females [48]; the vegetation on the lek itself often gets trampled down by the dancing displays [49]. Based on our field surveys and previous work, sharp-tailed grouse tend to choose hills as the site of their leks [50] but in relatively flat areas. By lekking in relatively flat areas, and choosing the few hills available, males can effectively create a stage to broadcast their displays to prospective females in the area. Future research could examine at a finer scale (i.e., less than the 1-km scale we used) whether male sharp-tailed grouse select hills with greater sight lines, or some other attribute, to facilitate the detectability of their displays to passing females.

Habitat suitability decreased once the 5 x 5-km landscape surrounding the lek had > 5% human disturbance. In a review of the effect of anthropogenic structures on various grouse species, lek site persistence was the portion of grouse life history most affected by anthropogenic structures [6]. More recent studies have also shown an avoidance by prairie grouse of developed areas [43]. Sharp-tailed grouse show high lek fidelity and as such may not ever fully abandon lek sites; however, their populations may decline over time at disturbed sites due to lower recruitment [51]. Alternatively, anthropogenic features may aid sharp-tailed grouse nest success by excluding potential predators displaced by the development [52]. Further research on specific anthropogenic features and their effects on sharp-tailed grouse lek occurrence is still needed.

There are several habitat features that could help to refine our modelling of sharp-tailed grouse lek occurrence. For example, we did not include vegetation height in our models even though it is typically important in many sharp-tailed grouse habitat suitability models [20, 53, 54]. Unfortunately, acquiring information on vegetation height is impractical at the province-wide scale and can be highly variable over time depending on land use and weather conditions. Finer-resolution environmental predictor layers would have also allowed us to also assess smaller habitat patches (e.g., shrub patches that might be smaller than the initial 30-m resolution of our landcover layer) and features (e.g., small roads, wells, powerlines) that have been demonstrated to be important in previous sharp-tailed grouse habitat selection studies [52]. This could explain why shrub cover, which is a key component of several sharp-tailed grouse habitat models [20, 55, 56] was less important in our models. Many of the shrub patches with lower height and density do not appear in the landcover layers we used and to our knowledge there are no other suitable shrub mapping products available in Saskatchewan.

### Management implications

The sharp-tailed grouse is a vital game bird in many jurisdictions, and ensuring that the necessary habitat is available to support it is integral to ensuring sustainable hunting opportunities [8, 57]. To do this effectively, wildlife managers must have access to tools that identify preferred and high quality grouse habitats [57]. Our lek occurrence model highlights the importance of maintaining large diverse grasslands for management of sharp-tailed grouse. In addition, within these large and intact grassland landscapes it is essential to maintain other various natural features. An area managed for sharp-tailed grouse and concurrent human use according to our model would need to retain a high proportion of grasslands (approximately 0.80), while limiting the amount of anthropogenic influences to less than 5% of this landscape. Using this HSI model, wildlife managers will be able to better predict sharp-tailed grouse habitat use and target conservation efforts to areas with high potential for the occurrence of breeding complexes, thereby more effectively managing the species at a large scale. Given their economic and cultural value [8], our research confirms that sharp-tailed grouse could be useful as an umbrella species for protecting northern grasslands and other species of conservation concern [58, 59] due to their concurrent need for relatively large patches of intact grasslands. However, future research should examine how habitat change could lead to changes in lek persistence or disappearance, and how the landscape features we identified as necessary for lek occurrence are related to habitat quality (survival, reproductive success) outside of the lekking season.
